# Safety Assessment on Serious Adverse Events of Targeted Therapeutic Agents Prescribed for RAS Wild-Type Metastatic Colorectal Cancer: Systematic Review and Network Meta-Analysis

**DOI:** 10.3390/ijerph19159196

**Published:** 2022-07-27

**Authors:** Yeo Jin Choi, Chang-Young Choi, Sandy Jeong Rhie, Sooyoung Shin

**Affiliations:** 1 Department of Pharmacy, College of Pharmacy, Kyung Hee University, Seoul 02447, Korea; 2 Department of Regulatory Science, Graduate School, Kyung Hee University, Seoul 02447, Korea; 3 Department of Internal Medicine, Ajou University Medical Center, Suwon 16499, Korea; changchoi1216@gmail.com; 4 Graduate School of Converging Clinical & Public Health, Ewha Womans University, Seoul 03670, Korea; sandy.rhie@ewha.ac.kr; 5 Graduate School of Pharmaceutical Sciences, College of Pharmacy, Ewha Womans University, Seoul 03760, Korea; 6 Department of Pharmacy, College of Pharmacy, Ajou University, Suwon 16499, Korea

**Keywords:** adverse events, bevacizumab, cetuximab, colorectal cancer, metastatic cancer, panitumumab, pharmacovigilance

## Abstract

Despite substantially elevated risk of serious adverse events (SAEs) from targeted therapy in combination with chemotherapy, comprehensive pharmacovigilance research is limited. This study aims to systematically assess SAE risks of commonly prescribed targeted agents (bevacizumab, cetuximab, and panitumumab) in patients with rat sarcoma viral oncogene homolog (RAS) wild-type metastatic colon cancer. Keyword searches of Cochrane Library, Clinical Key and MEDLINE were conducted per PRISMA-NMA guidelines. Frequentist network meta-analysis was performed with eight randomized controlled trials to compare relative risk (RR) of 21 SAE profiles. The risks of hematological, gastrointestinal, neurological SAE were insignificant among targeted agents (*p* > 0.05). The risk of serious hypertension was substantially elevated in bevacizumab-based chemotherapy (*p* < 0.05), whereas panitumumab-based chemotherapy had markedly elevated risk of serious thromboembolism (RR 3.65; 95% CI 1.30–10.26). Although both cetuximab and panitumumab demonstrated increased risk of serious dermatological and renal toxicities, panitumumab-based chemotherapy has relatively higher risk of skin toxicity (RR 15.22; 95% CI 7.17–32.35), mucositis (RR 3.18; 95% CI 1.52–6.65), hypomagnesemia (RR 20.10; 95% CI 5.92–68.21), and dehydration (RR 2.81; 95% CI 1.03–7.67) than cetuximab-based chemotherapy. Thus, further studies on risk stratification and SAE management are warranted for safe administration of targeted agents.

## 1. Introduction

The incidence of colorectal cancer (CRC) is increasing each year, with 1.9 million new diagnoses worldwide in 2020 [1]. CRC is the second most common cause of cancer-related mortality, accounting for 935,000 mortalities worldwide [2]. Although early diagnosis of CRC is suggested with better prognosis of the disease, about 25% of localized CRC patients have increased risk for metastatic progression, and ultimately more than 50% of CRC patients develop advanced or metastatic CRC [2,3]. The treatment modalities for metastatic CRC primarily involve surgical resection and medication management with chemotherapy and targeted immunotherapy, and considering that most metastatic CRC is often unresectable, appropriate medication management cannot be neglected to improve patient outcomes [4].

Despite numerous studies on the advances of treatment modalities such as chemoradiation and gene therapy in metastatic CRC patients [5,6,7,8,9], the National Comprehensive Cancer Network (NCCN) guidelines recommend administration of targeted therapeutic agents with classical backbone chemotherapy regimens such as CAPEOX (capecitabine and oxaliplatin), FOLFIRI (5-fluorouracil, leucovorin, and irinotecan), FOLFOX (5-fluorouracil, leucovorin, and oxaliplatin), or FOLFOXIRI (5-flurouracil, leucovorin, oxaliplatin, and irinotecan) for unresectable metastatic CRC [4]. The two most commonly recommended biological classes of targeted agents include vascular endothelial growth factor (VEGF) antibodies that inhibit cancer angiogenesis and epidermal growth factor receptor (EGFR) inhibitors that block cancer proliferation [4]. Among various VEGF antibodies, bevacizumab is the most preferred targeted therapeutic agent for conversion therapy while EGFR inhibitors, cetuximab and panitumumab, are recommended only in patients with rat sarcoma viral oncogene homolog (RAS) wild-type left-sided metastatic CRC, suggesting a significant impact of genetic variability on patient outcomes [4,10].

Previous studies on clinical effects of these agents, in terms of tumor responses and survival rates, demonstrated considerably higher efficacy of EGFR inhibitors than VEGF antibodies in left-sided colon cancer [11]. Nevertheless, identifying the optimal chemotherapeutic regimen is still a challenge as various factors including age, gender, genetic variability, or co-morbidities affect patient responses to chemotherapy [12]. Moreover, adverse events (AEs) from medication management may also impede appropriate treatment, negatively affecting patient prognosis as well as quality of life [13,14]. Compared to traditional chemotherapy, targeted therapeutic agents are generally well-tolerated [15]. However, as the majority of metastatic CRC patients are administered with classic chemotherapy regimens in combination with targeted therapeutic agents, these patients tend to be more susceptible to therapy-induced AEs [16]. Nevertheless, systematic analyses on targeted biologics-associated AEs in metastatic CRC patients are still limited. In consideration of approximately 44.5% of AEs from chemotherapy being classified as serious adverse effects (SAE) [17], there is an urgent need for safety analyses of different combination regimens involving administration of both targeted agents and classic chemotherapy in metastatic CRC patients. Hence, the purpose of this systematic review and network meta-analysis is to comprehensively assess the safety of targeted biologic agents (bevacizumab, cetuximab, and panitumumab) that are commonly prescribed as first-line agents in patients with RAS wild-type metastatic CRC [4], in terms of SAE incidences of 21 AE profiles in six different organ systems, and to provide guidance on the selection of targeted therapeutic agents based on SAE profiles.

## 2. Materials and Methods

### 2.1. Data Sources and Search Strategy

This study was performed in accordance with the Preferred Reporting Items for Network Meta-Analyses (PRISMA-NMA) guidelines [18]. MEDLINE (PubMed), the Cochrane Central Register of Controlled Trials (CENTRAL), and ClinicalKey were searched from inception to February 2022 for relevant clinical studies. The initial database search involved a combination of Medical Subject Headings (MeSH) and keywords in the title/abstract: ‘colorectal cancer’, ‘colon cancer’, ‘bevacizumab’, ‘cetuximab’, ‘panitumumab’, or ‘metastatic cancer’. The prespecified search filters include ‘clinical trials’, ‘humans’ and ‘English’. We manually searched the references of eligible articles to identify additional studies for systematic review and network analysis.

### 2.2. Study Selection and Data Extraction

Two reviewers independently screened the titles and abstracts of all studies identified from the initial searches for eligibility. Any disagreements on eligibility of studies were further discussed until consensus was reached. The eligibility of studies was determined by prespecified inclusion criteria: (1) head-to-head randomized controlled trials (RCTs) evaluating safety of biological targeted agents with classic chemotherapy, (2) patients with RAS wild-type metastatic CRC, (3) studies evaluating SAEs between two different targeted agents, in metastatic CRC patients on the same backbone chemotherapy regimen including CAPEOX, FOLFOX, FOLFIRI, FOFOXIRI, or irinotecan, (4) administration of the intervention or the comparator as first- or second-line therapy, and (5) studies published in English. Duplicate studies, review articles, case reports, conference abstracts, study protocols, commentaries, editorials, proceedings, and studies without available full-texts were excluded from the analysis. Any studies evaluating investigational products without approval from the Food and Drug Administration (FDA) were excluded from the analysis. The following information was extracted from eligible studies: study characteristics (authors, study design, year of publication, study periods, and study region), study population (inclusion and exclusion criteria and number of patients assigned to each treatment arm), study interventions and comparators (medication names, dosages, types of backbone chemotherapy) and safety outcomes. The safety outcomes were classified per common terminology criteria for Adverse Events (CTCAE) version 5.0, and SAE was defined as any AEs classified as grade 3 (severe AEs) or 4 (life-threatening or disabling AEs) per CTCAE guidelines [19]. The SAEs were further classified according to the affected physiological systems, including the hematological, gastrointestinal (GI), neurological, dermatological, renal and cardiovascular (CV) systems. The PICOS (patient, intervention, comparator, outcomes, study design) is summarized in Table 1.

### 2.3. Assessment of Bias Risk and Evidence

The quality assessment of the studies included in this analysis was performed based on Cochrane Risk of Bias version 2.0 (RoB2) tool [20,21], and the studies were scored as low, some concern (unclear), or high risk of bias in the following aspects: bias due to randomization, bias due to deviation from intended intervention, bias due to missing data, bias due to outcome measurement, bias due to selection of reported results, and overall bias. Any disagreements on the study quality assessment were discussed until consensus was reached.

### 2.4. Statistical Methods

Pooled traditional pair-wise analysis on the safety outcomes were evaluated using R (version 4.1.0). Frequentist network meta-analyses were performed to combine direct and indirect effects and simultaneously compare SAE incidences of three different targeted therapeutic agents in combination with chemotherapy for management of metastatic CRC against a control group generated by the network [22,23]. The SAEs were analyzed with relative risks (RR), and 95% confidence interval (CIs) were estimated to approximate the risk of each SAE profile in metastatic CRC patients receiving targeted therapeutic agent with backbone chemotherapy. Reverse percentages were calculated for any results reported in terms of percentage (%) in the original articles. I^2^ index was utilized to assess heterogeneity across the studies [24], and the Mantel–Haenszel fixed-effect model was used to analyze outcomes with low-heterogeneity (I^2^ < 50%), and the random-effect model was applied to analyze outcomes with high heterogeneity (I^2^ > 50%). Cochran’s Q was utilized to assess the extent of inconsistency, and any *p*-value < 0.05 was considered as significant [24,25]. The netrank function was utilized to evaluate the ranking of relative safety of targeted therapy agents in combination with classic CRC chemotherapy for the treatment of metastatic CRC: the larger the *p*-value, the better the rank of the intervention, and *p*-value is an equivalent to the Surface Under the Cumulative Ranking (SUCRA) score [26]. Subgroup analysis was performed to analyze differences in SAE risks of each targeted agent when prescribed as first- or second-line metastatic CRC treatment. All *p*-values were estimated by two-sided tests and *p*-values < 0.05 were considered statistically significant.

## 3. Results

### 3.1. Study Search and Selection

**Figure 1:**
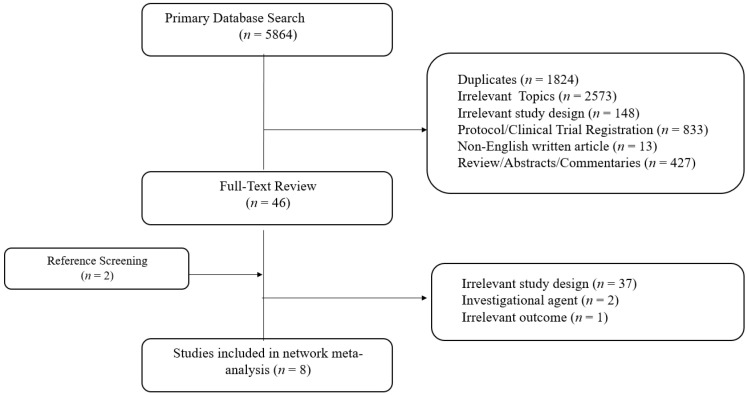
PRISMA plot.

The study search and selection results per PRISMA-NMA guidelines are described in Figure 1. The primary search of ClinicalKey, the Cochrane Library, and MEDLINE (PubMed) yielded 5864 studies. A total of 48 studies were eligible for full-text reviews after the exclusion of duplicates, irrelevant studies, including those with irrelevant study designs, medications and populations, reviews, study protocols or clinical trial registrations, and abstracts including conference abstracts. After full-text review, a total of eight studies with 2685 patients diagnosed with RAS wild-type metastatic CRC were included for network meta-analysis: 1270 patients on bevacizumab plus backbone chemotherapy, 1060 patients on cetuximab plus backbone chemotherapy and 355 patients on panitumumab plus backbone chemotherapy.

### 3.2. Eligible Study Characteristics

**Figure 2:**
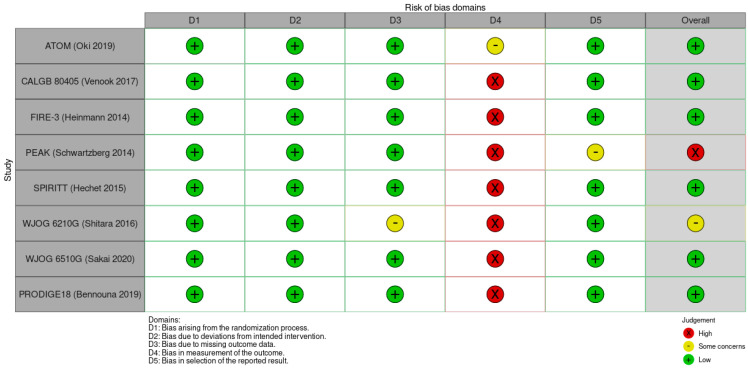
Study quality assessment per RoB 2.

**Figure 3:**
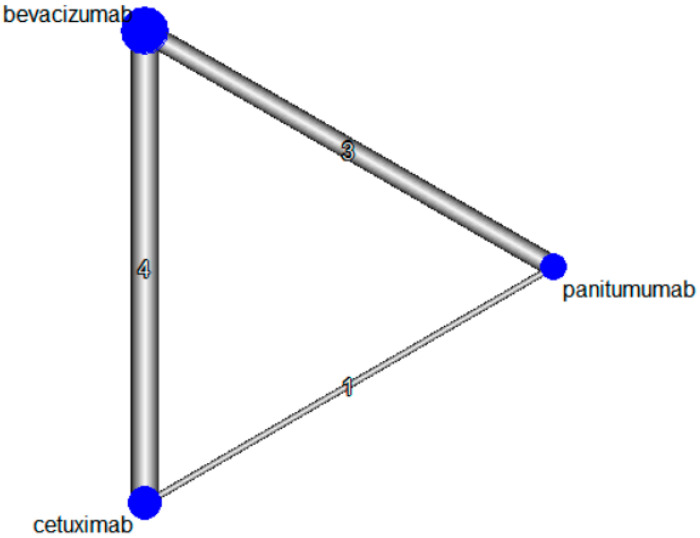
Network plot of included studies. Diameters of the blue circle represent the proportion of patients included in the analysis, and thickness of the lines is weighted by the number of the studies comparing two interventions.

The characteristics of the eligible studies for network meta-analysis and bias assessments are summarized in Table 2 and Figure 2, respectively. Four studies evaluated the safety of first-line targeted therapeutic agents with backbone chemotherapy [27,28,29,30], three studies evaluated the safety of second-line targeted therapeutic agents with backbone chemotherapy in metastatic CRC patients who failed previous chemotherapy combined with bevacizumab [31,32,33], and one study evaluated the safety of second-line targeted therapeutic agents with backbone chemotherapy in metastatic CRC patients who failed chemotherapy without targeted therapeutic agent [34]. The backbone chemotherapy regimens were FOLFOX [27,28,30,31], FOLFIRI [28,29,31,32,33], and irinotecan [34]. The *p*-value of Cochran Q’s statistic was > 0.05 for all safety outcomes, indicating low risk of inconsistency. The network plot for the included studies is shown in Figure 3.

### 3.3. Safety Outcomes 

**Figure 4:**
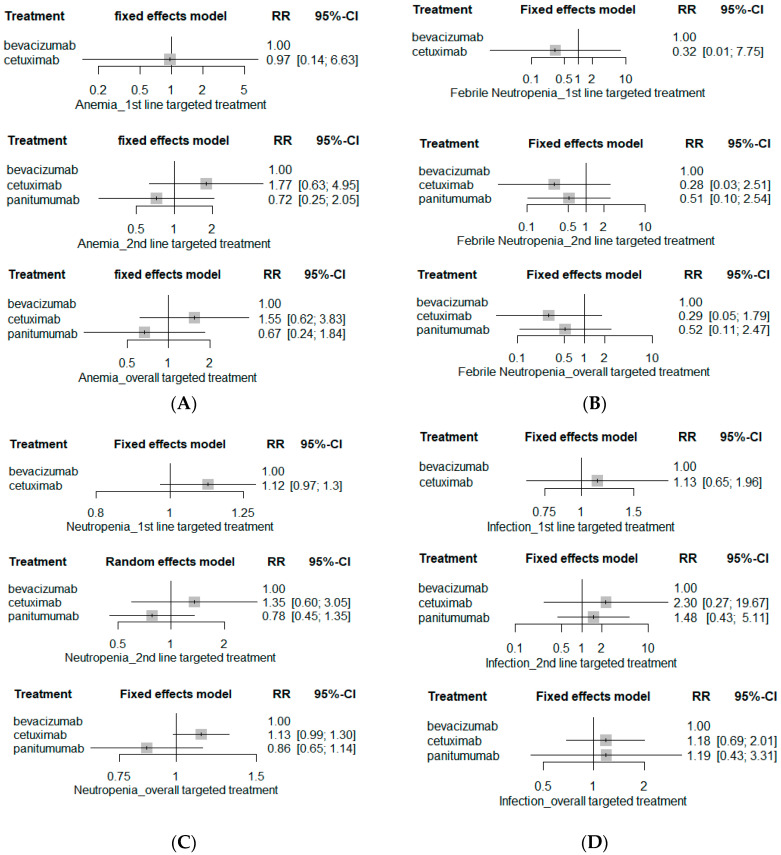

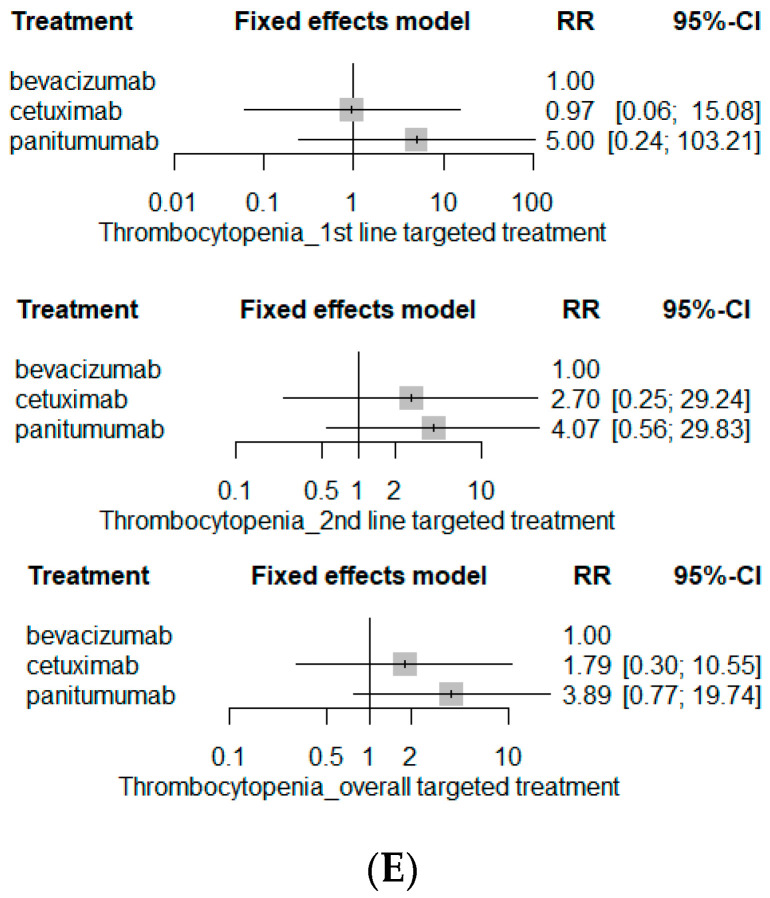
Hematological SAE risks. (**A**) Anemia; (**B**) febrile neutropenia; (**C**) neutropenia; (**D**) infection; and (**E**) thrombocytopenia.

**Figure 5:**
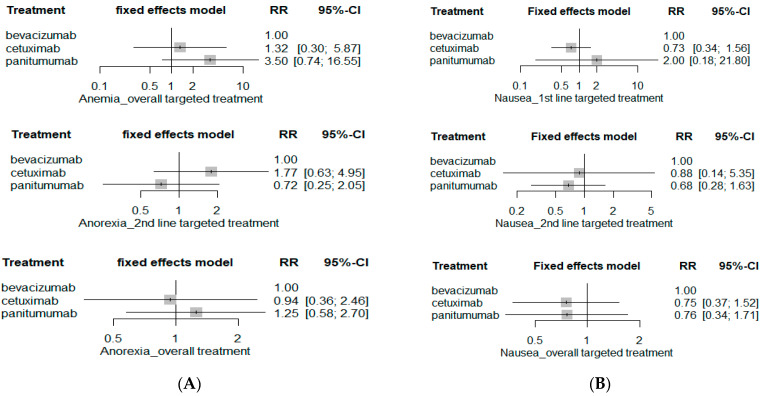

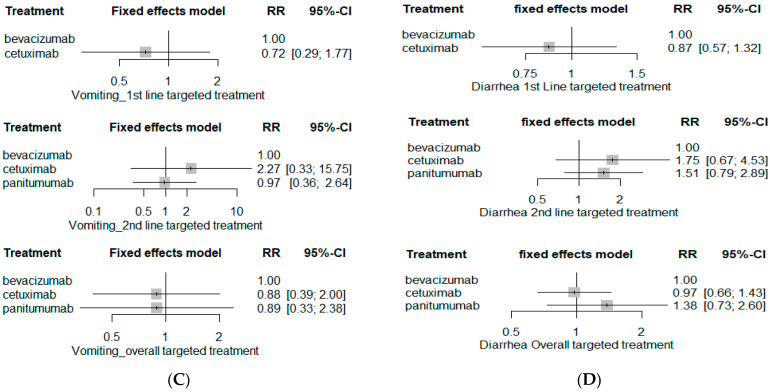
GI SAE risks. (**A**) Anorexia; (**B**) nausea; (**C**) vomiting; and (**D**) diarrhea.

**Figure 6:**
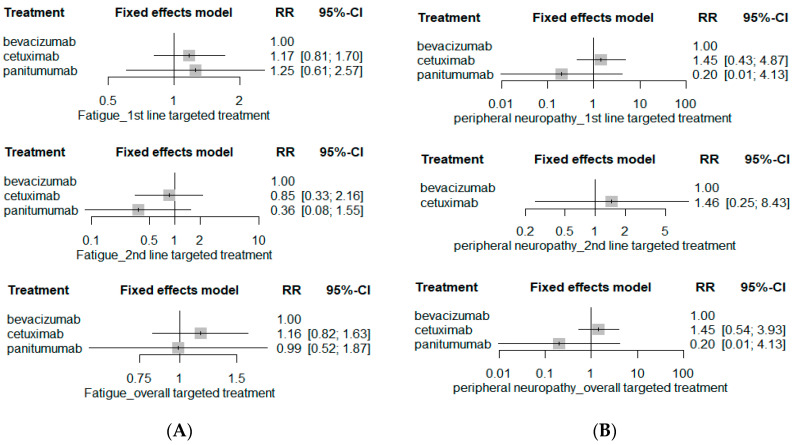
Neurological SAE risk. (**A**) Fatigue; and (**B**) peripheral neuropathy.

The risk of hematological SAE including anemia, febrile neutropenia, neutropenia, infection and thrombocytopenia was comparable among bevacizumab-based chemotherapy, cetuximab-based chemotherapy, and panitumumab-based chemotherapy (Figure 4). Moreover, the risks of serious GI-related AEs such as anorexia, nausea, vomiting, and diarrhea as well as neurological SAEs including fatigue and peripheral neuropathy were similar among bevacizumab-, cetuximab-, and panitumumab-based chemotherapy (Figure 5 and Figure 6). The network plots of direct, indirect and estimated summary of all safety outcomes and safety rankings of targeted therapeutic agents for each safety outcome are summarized in the Supplementary Materials (Figures S1–S6 and Table S1).

**Figure 7:**
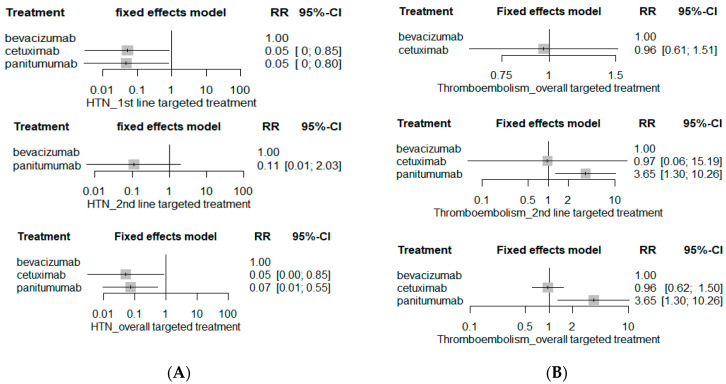
Cardiovascular SAE risks. (**A**) Hypertension; and (**B**) thromboembolism.

The risk of serious hypertension (HTN) was significantly higher in the bevacizumab-based chemotherapy group than either cetuximab-based chemotherapy (RR 0.05; 95% CI 0.00–0.85) or panitumumab-based chemotherapy (RR 0.07; 95% CI 0.01–0.55) (Figure 7). However, the risk of serious HTN was statistically insignificant between second-line bevacizumab-based chemotherapy and second-line panitumumab-based chemotherapy (RR0.11; 95% CI 0.01–2.03), though RR is fairly high with panitumumab-based chemotherapy. On the other hand, the risk of serious thromboembolism was substantially higher in panitumumab-based chemotherapy (RR 3.65; 95% CI 1.30–10.26) than cetuximab- (RR 0.96; 95% CI 0.62–1.50) or bevacizumab-based chemotherapy (RR 1, reference), and the RR of serious thromboembolism of panitumumab-based chemotherapy was 3.79 (95% CI 1.23–11.67) when compared with cetuximab-based chemotherapy (data not shown).

**Figure 8:**
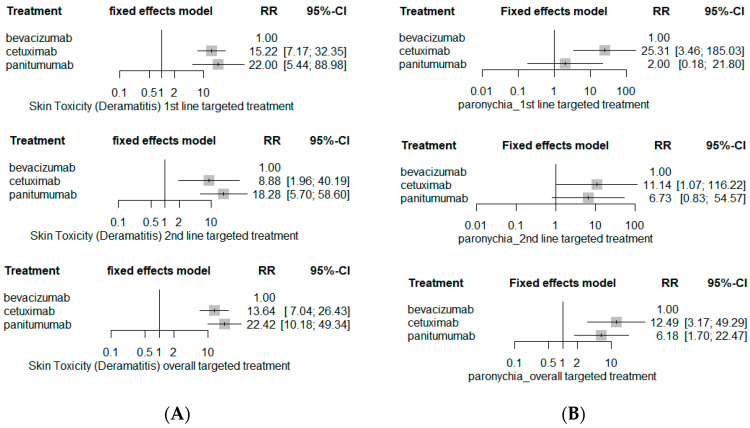

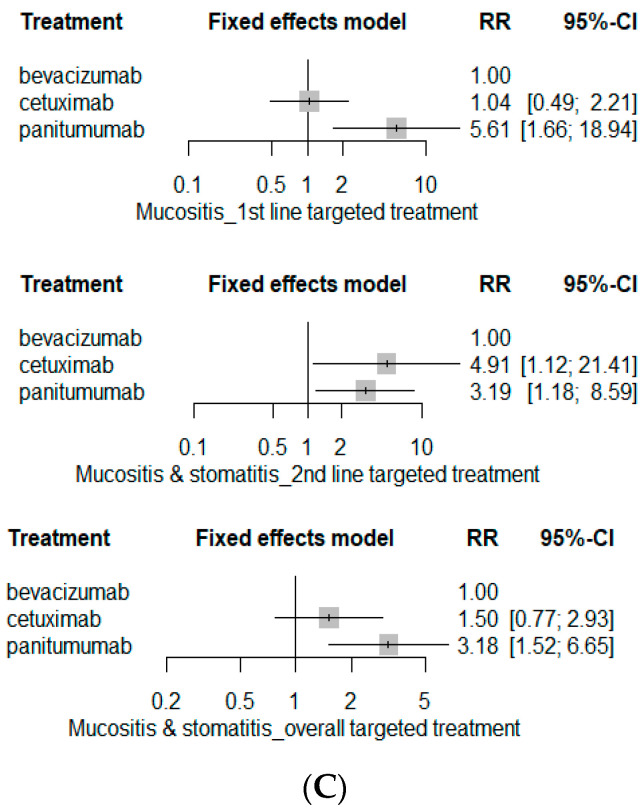
Dermatological SAE risks. (**A**) Rash (skin toxicity); (**B**) paronychia, and (**C**) mucositis.

The risk of serious skin toxicities involving rash or dermatitis was substantially higher with EGFR inhibitor-based chemotherapy (Figure 8): cetuximab-based chemotherapy (RR 13.64; 95% CI 7.04–26.43) or panitumumab-based chemotherapy (RR 22.42; 95% CI 10.18–49.34). The risk of serious paronychia was also markedly higher in EGFR-inhibitor-based chemotherapy (RR 12.49; 95% CI 3.17–49.29 for cetuximab-based chemotherapy and RR 6.18; 95% CI 1.70–22.47 for panitumumab-based chemotherapy), but subgroup analyses revealed contrary results; cetuximab-based chemotherapy markedly elevated the risk of paronychia (RR 25.31; 95% CI 3.46–185.03 for first-line and RR 11.14; 95% CI 1.07–116.22 for second-line) whereas panitumumab-based chemotherapy had statistically insignificant paronychia risk when compared to bevacizumab-based chemotherapy (RR 2.00; 95% CI 0.18–21.80 for first-line targeted treatment; RR 6.73; 95% CI 0.83–54.57 for second-line targeted treatment). On the other hand, panitumumab-based chemotherapy had the greatest risk of serious mucositis/stomatitis (RR3.18; 95% CI 1.52–6.65).

**Figure 9:**
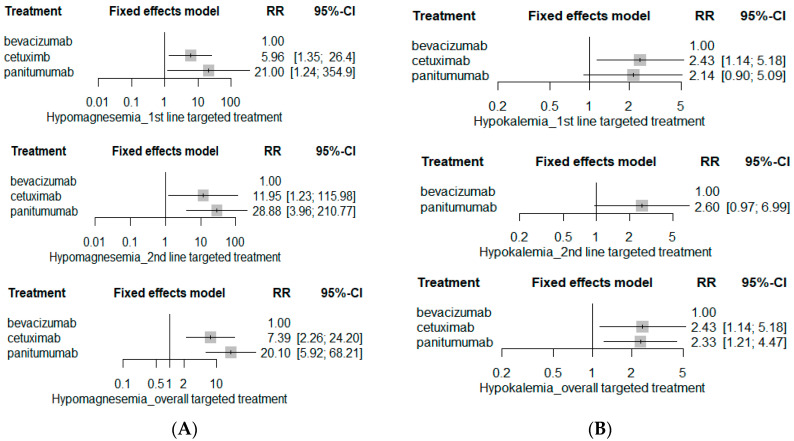

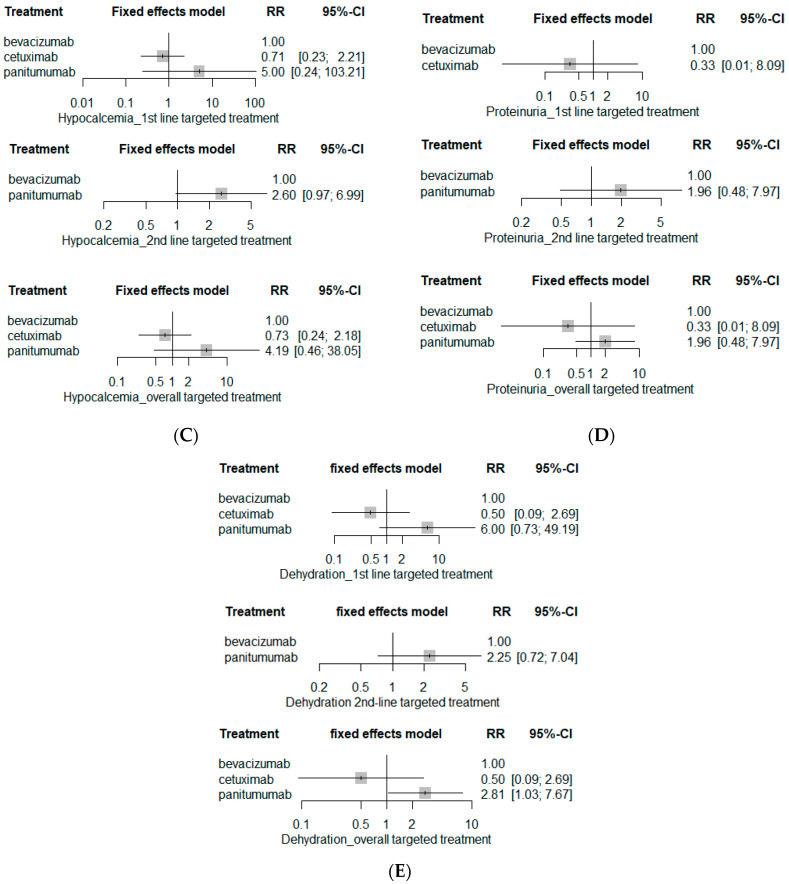
Renal SAE risk. (**A**) Hypomagnesemia; (**B**) hypokalemia; (**C**) hypocalcemia; (**D**) proteinuria; and (**E**) dehydration.

EGFR inhibitors including cetuximab and panitumumab markedly increased SAE risks pertaining to renal systems (Figure 9). Both cetuximab and panitumumab substantially elevated the risks of serious electrolyte abnormalities including hypomagnesemia (RR 7.39; 95% CI 2.26–24.20 for cetuximab-based chemotherapy; and RR 20.10; 95% CI 8.92–68.21 for panitumumab-based chemotherapy) and hypokalemia (RR 2.43; 95% CI 1.14–5.18 for cetuximab-based chemotherapy; RR 2.33; 95% 1.21–4.47 for panitumumab-based chemotherapy), whereas risks of serious hypocalcemia and proteinuria were insignificant among three targeted agents. The risk of serious dehydration was the highest in patients receiving panitumumab-based chemotherapy (RR 2.81; 95% CI 1.03–7.67).

## 4. Discussion

Combining targeted biologics with classic CRC chemotherapy, such as CAPEOX, FOLFOX, FOLFIRI or FOLOXIRI, is effective in inhibiting tumor progression but at the cost of increased risk of AEs [4,35,36]. Despite their substantial influence on the deteriorating quality of life of those undergoing chemotherapy, AE prevention can be occasionally underemphasized in patient care. Such risk becomes higher in metastatic cancer management since tumor size reduction as well as tumor progression inhibition are considered more critical to improve the markedly high mortality rates in these patients [3]. In this study, we performed comparative safety assessment of three preferably prescribed first-line targeted therapeutic agents on 21 SAE (Grade 3 and 4 per CTCAE) profiles in six different organ systems, including hematological, GI, CV, neurological, dermatological and renal systems, when administered concomitantly with backbone chemotherapy in metastatic CRC patients. Our network meta-analysis demonstrated insignificant risks of hematological, GI and neurological SAEs among bevacizumab-, cetuximab-, and panitumumab-based chemotherapy. However, substantial differences in SAE profiles among these agents were assessed in CV, dermatological, and renal systems.

Similar to the results from a previous meta-analysis on the tolerability on SAE of first-line bevacizumab and cetuximab for RAS wild-type metastatic CRC patients [37], the highest risk of serious HTN was observed in bevacizumab-based chemotherapy (RR 1.00; reference) over cetuximab- (RR 0.05; 95% CI 0.00–0.85) and panitumumab-based chemotherapy (RR 0.07; 95% CI 0.01–0.55); the RR of HTN was insignificant between second-line bevacizumab- and panitumumab-based chemotherapy. However, caution is advised with the interpretation of safety assessment results of second-line targeted treatment as it was evaluated with only one RCT [32], which demonstrated 4 and 0 cases of serious HTN (Grade 3 or 4 per CTCAE) in patients on bevacizumab- and cetuximab-based chemotherapy, respectively. 

The risk of serious thromboembolism, on the other hand, was substantially elevated with panitumumab-based chemotherapy (RR 3.65; 95% CI 1.30–10.26) when compared to bevacizumab- (RR 1.00, reference) and cetuximab-based chemotherapy (RR 0.96; 95% CI 0.62–1.50). According to previous studies [38], patients on EGFR inhibitors, either cetuximab or panitumumab, are at higher risk of thromboembolism, especially with venous thromboembolism (VTE), than those not treated with EGFR inhibitors. Although data on head-to-head safety comparison between cetuximab and panitumumab are still sparse, this study provided evidence on higher risk of serious thromboembolism with panitumumab-based chemotherapy than cetuximab-based chemotherapy (RR 3.79; 95% CI 1.23–11.67, data not shown). Cancer itself plays a role as a crucial risk factor for VTE due to cancer-induced hypercoagulability [39], and the VTE incidence surges with repeated exposure to chemotherapy [40,41,42]. Furthermore, certain anti-cancer agents including targeted agents substantially increase thrombosis risks [43]. Although the exact mechanism of thrombosis induced by EGFR inhibitors is yet to be elucidated, studies suggest that subsequent antiangiogenic effects involving reduction in angiogenic growth factors via EGFR blockade may be responsible for thrombosis events [44,45], which also implies that bevacizumab, an agent that directly inhibits angiogenesis, may also potentiate VTE risks. Despite considerably lower risk of serious VTE with bevacizumab when compared to panitumumab, studies still report controversial VTE risks of bevacizumab [46,47]. Thus, judicious administration of targeted therapy-combined chemotherapy accompanied by appropriate VTE prophylaxis are strongly encouraged in metastatic CRC patients, and further studies on the mechanism and definite VTE risk associated with each targeted agent are warranted to improve patient safety. 

Skin toxicities, including acneiform skin rash and nail disorders, are well-known AEs of EGFR inhibitors [35], and previous meta-analysis from our research group also suggested higher risk of skin toxicities with cetuximab over bevacizumab [37]. This study revealed a significantly greater risk of serious dermatitis or paronychia with EGFR inhibitors, cetuximab and panitumumab. However, interesting trends were assessed regarding serious mucositis; statistically higher risk of serious mucositis was observed with panitumumab-based chemotherapy (RR 3.18; 95% CI 1.52–6.65) when referenced with bevacizumab-based chemotherapy, whereas the risk of serious mucositis was insignificant among three targeted agents when referenced with cetuximab (RR 0.59; 95% CI 0.27–1.28 for bevacizumab-based chemotherapy, and RR 1.92; 95% CI 0.70–5.29 for panitumumab-based chemotherapy; data not shown). Mucositis is a common AE noticed in cancer patients on chemotherapy and is associated with poor quality of life [48,49]. Evidence suggests that the incidence of mucositis increases with concomitant administration with EGFR inhibitors such as cetuximab and panitumumab [50]. Moreover, consistent with our study results, a retrospective cohort study revealed a higher rate of mucositis in patients on panitumumab than those on cetuximab [50]. Nonetheless, uncertainty remains about whether the risk of serious mucositis is higher with panitumumab than with cetuximab, but careful monitoring and management of mucositis are warranted especially in panitumumab-treated patients due to potential risks. 

Electrolyte disorders including hypomagnesemia and hypokalemia are other AEs commonly observed in patients receiving EGFR inhibitor treatment. The network analysis revealed significantly higher incidence of serious hypomagnesemia and hypokalemia in cetuximab- and panitumumab-based chemotherapy, whereas the risk of serious hypocalcemia was insignificant among targeted therapeutic agents. Although some studies suggest that electrolyte disturbances, especially hypomagnesemia, may be associated with better clinical outcomes in terms of survival, appropriate management guidelines on electrolyte disturbances have yet to be established [51]. Furthermore, despite the significant dehydration risk associated with cancer and chemotherapy, recommendations on hydration therapy in metastatic cancer patients remain insufficient. Hence, considering that most cancer patients are already susceptible to electrolyte disturbances as well as dehydration secondary to cancer, further studies on risk stratification with appropriate management measures are warranted to improve patient outcomes from EGFR inhibitor therapy [52]. 

Based on the health-related quality of life study, worsening quality of life was observed in patients during chemotherapy [53], and AEs such as nausea, peripheral neuropathy, peripheral edema, and loss of appetite also serve as negative factors [54]. Despite a significant impact of AEs on patient prognosis and outcome, studies comprehensively investigating AE risks of targeted therapeutic agents are limited. Thus, this study was designed to comprehensively analyze 21 individual SAE profiles of three most preferably prescribed targeted therapeutic agents including bevacizumab, cetuximab and panitumumab in RAS wild-type metastatic CRC patients. To the best of our knowledge, this is the first network meta-analysis comprehensively investigating SAEs of targeted therapeutic agents prescribed with a classic chemotherapy regimen, and this study presents some novel findings: the highest risk of serious thromboembolism with panitumumab-based chemotherapy when compared to bevacizumab- and cetuximab-based chemotherapy. Furthermore, panitumumab-based chemotherapy was associated with relatively higher risk of serious dermatological toxicities involving skin toxicities, acneiform rash, and mucositis and renal toxicities such as hypomagnesemia and dehydration than the cetuximab-based chemotherapy. Panitumumab has been reported to have superior efficacy [34] in terms of prolonging overall survival and progression-free survival, compared to cetuximab, and economic analyses revealed lower projected cost with panitumumab in metastatic CRC patients, advocating a preference of panitumumab over cetuximab as first-line therapy [55,56]. However, considering these studies included infusion reaction as the only AE in the analysis, the risk versus benefits of first-line panitumumab-based chemotherapy should be reassessed. Moreover, further studies investigating the impact of higher SAE risks of panitumumab-based chemotherapy on patient prognosis and economic values are warranted. 

This study has several limitations. First, the study designs and outcome measurements were different among the studies included in the analysis, subsequently increasing heterogeneity across the studies. Moreover, due to the nature of cancer-related RCTs, which recruit vulnerable patient populations, the number of patients in each group was relatively small to perform subgroup analysis, and this may decrease the robustness of the study results. Moreover, subsequent analyses on the impact of various factors including age, gender, and comorbidities on AE risks of targeted therapeutic agents are limited at this point. Nonetheless, this study has strong external validity as patients received different backbone chemotherapy regimens including FOLFOX, FOLFIRI, and irinotecan. Additionally, the meaningful aspect of this study is that this study provides evidence on comprehensive network analysis of SAE profiles of the most commonly prescribed targeted therapeutic agents in metastatic CRC patients who are highly susceptible to increased risk of AEs from administration of multiple cytotoxic agents, thereby providing guidance on the selection of targeted therapeutic agents as well as AE management plans based on the SAE profiles. Nevertheless, further research on AE mechanism as well as risk factors in terms of patient characteristics associated with SAE of each targeted therapeutic agent is recommended to improve patient prognosis as well as quality of life. 

## 5. Conclusions

The risk of SAEs in hematological, neurological and GI system are statistically insignificant among bevacizumab-, cetuximab-, and panitumumab-based chemotherapy. The risk of serious HTN is the highest in the bevacizumab-based chemotherapy group. However, panitumumab-based chemotherapy has the highest risk of serious thromboembolism than cetuximab- and bevacizumab-based chemotherapy. Administration of the EGFR inhibitors, cetuximab and panitumumab, substantially elevated the risk of dermatological and renal SAEs. However, the risks of skin toxicities, mucositis, hypomagnesemia and dehydration are relatively higher in panitumumab-based chemotherapy than cetuximab-based chemotherapy. Hence, further studies investigating the mechanisms and risk factors associated with these SAEs to provide risk-stratified guidance on targeted agents to ensure patient safety. 

## Figures and Tables

**Table 1:** PICOS criteria for study selection.

Parameter	Criteria
**P: Patients**	Patients diagnosed with RAS wild-type metastatic CRC who administered the intervention or the comparator as first or second-line treatment
**I: Intervention**	Bevacizumab + classical CRC chemotherapy
**C: Comparison**	Cetuximab + classical CRC chemotherapy Panitumumab + classical CRC chemotherapy
**O: Outcomes**	SAE (Grade 3–4) per CTCAE **Hematological SAE:** anemia, febrile neutropenia, neutropenia, infection, thrombocytopenia **CV SAE:** hypertension, thromboembolism **Dermatological SAE:** skin toxicity (dermatitis or rash), paronychia, mucositis/stomatitis **GI SAE:** anorexia, nausea, vomiting, diarrhea **Renal SAE:** electrolyte abnormalities, proteinuria, dehydration **Neurological SAE:** peripheral neuropathy and fatigue
**S: Study design**	Randomized Controlled Trials

Abbreviation: CTCAE: common terminology criteria for adverse events; CV: cardiovascular; CRC: colorectal cancer; GI: gastrointestinal; RAS: rat sarcoma viral oncogene homolog; SAE: serious adverse effects.

**Table 2:** Study characteristics of included studies.

Study Name	Study Duration	Country	Study Design	Patient Population	Intervention	Comparator	Backbone Chemotherapy
***First-Line (Treatment naïve)***							
**ATOM** **(Oki2019)**	May 2013–April 2016	Japan	Multicenter, randomized phase II study	Patients aged between 20 and 80 years with liver-limited metastases from wild-type (K) RAS CRC	Bevacizumab (5 mg/kg) (*n* = 57)	Cetuximab (400 mg/m^2^ first dose followed by 2400 mg/m^2^ on Day 1 through Day 2) (*n* = 59)	mFOLFOX6
**CALGB 80,405 (Venook 2017)**	November 2005–March 2012	United States and Canada	Multicenter, randomized, phase III study	Patients aged ≥ 18 years with previously untreated advanced or metastatic colorectal cancer whose tumors were KRAS WT	Bevacizumab (5 mg/kg) (*n* = 559)	Cetuximab (400 mg/m^2^ followed by 250 mg/m^2^) (*n* = 578)	mFOLFOX6 or FOLFIRI
**FIRE-3 (Heinemann 2014)**	23 January 2007–19 September 2012	Germany, Austria	Randomized, open-label, Phase 3 trial	Age 18–75 years with stage IV, histologically confirmed adenocarcinoma of the colon or rectum, ECOG performance status of 0–2, an estimated life expectancy of greater than 3 months and adequate organ function, and no surgery within 4 weeks before the study	Bevacizumab (5 mg/kg) (*n* = 295)	Cetuximab (400 mg/m^2^ on Day 1 and 250 mg/m^2^ weekly) (*n* = 297)	FOLFIRI
**PEAK (Schwartzberg 2014)**	April 2009 and December 2011		Phase II multicenter, open-label, randomized two-arm study	Age ≥ 18 years, ECOG performance of 0 or 1, histologically or cytologically confirmed metastatic adenocarcinoma of the colon or rectum with unresectable metastatic disease, WT KRAS exon2 (codons 12 and 13)	bevacizumab 5 mg/kg every two weeks (*n* = 143)	panitumumab 6 mg/kg every 2 weeks (*n* = 142)	mFOLFOX6
***Second-Line***							
**SPIRITT** **(Hechet 2015)**	November 2006–December 2010	United States	Randomized, phase II	Age ≥ 18, ECOG performance score of 0 or 1, had histologically or cytologically confirmed metastatic adenocarcinoma of the colon or rectum. Failed previous first-line oxaliplatin-based chemotherapy with bevacizumab	Bevacizumab 5 mg/kg (*n* = 91)	Panitumumab 6 mg/kg (*n* = 91)	FOLFIRI
**WJOG 6210G** **(Shitara 2016)**	April 2011 and Febrary 2014	Japan	Randomized phase II trial	Histopathologically proven unresectable distant metastatic or locally advanced colorectal adenocarcinoma, presence of radiographically confirmed or clinically diagnosed disease progression during or within 3 months after the last dose of first-line chemotherapy containing fluoropyrimidine, oxaliplatin, and bevacizumab, and confirmation of WT KRAS exon2 (codon 12 or 13)	Bevacizumab 5 mg/kg (*n* = 60)	Panitumumab 6 mg/kg (*n* = 61)	FOLFIRI
**WJOG6510G** **(Sakai 2020)**	December 2011–September 2014	Japan	Open-label, randomized, multicenter, phase II study	Histopathologically confirmed unresectable mCRC; failure of prior chemotherapy with fluorouracil-, oxaliplatin, and irinotecan-based therapy, wild-type KRAS exon2 based on the test at the local institution; age ≥ 20 years; PS ≤ 2	Panitumumab 6 mg/kg (*n* = 61)	Cetuximab 400 mg/m^2^ followed by 250 mg/m^2^ (*n* = 59)	Irinotecan
**PRODIGE18** **(Bennouna 2019)**	14 December 2010–5 May 2015	France	Prospective, open-label, multicenter, randomized phase 2 trial	Patients 18 years of age or older, with Eastern Cooperative Oncology Group performance status of 0 or 1, histologically or cytologically proven mCRC, and with WT KRAS exon2. First-line treatment of mCRC with bevacizumab plus fluoropyrimidines and irinotecan or oxaliplatin	Bevacizumab(5 mg/kg) (*n* = 65)	Cetuximab (500 mg/m^2^) (*n* = 67)	FOLFIRI mFOLFOX

Abbreviations: CRC: colorectal cancer; ECOG: Eastern Cooperative Oncology Group; FOLFIRI: 5-fluorouracil, leucovorin, and irinotecanmFOLFOX: modified regimen of 5-fluorouracil, leucovorin, and oxaliplatin; WT: wild-type.

## Data Availability

The data presented in this study are available in the article or Supplementary Materials cited in the references.

## Supplementary Materials

The following supporting information can be downloaded at: https://www.mdpi.com/article/10.3390/ijerph19159196/s1, Table S1. Safety ranking of biological targeting therapeutic agents; Figure S1. Network plot of direct, indirect, network estimates of hematological SAE. (A) Anemia; (B) febrile neutropenia; (C) neutropenia; (D) infection; and (E) thrombocytopenia; Figure S2. Network plot of direct, indirect, network estimates of GI SAE. (A) Anorexia; (B) nausea; (C) vomiting; and (D) diarrhea; Figure S3. Network plot of direct, indirect, network estimates of neurological SAE. (A) Fatigue and (B) peripheral neuropathy; Figure S4. Network plot of direct, indirect, network estimates of CV SAE. (A) HTN and (B) thromboembolism; Figure S5. Network plot of direct, indirect, network estimates of dermatological SAE. (A) Rash (skin toxicity); (B) paronychia; and (C) mucositis; Figure S6. Network plot of direct, indirect, network estimates of renal SAE. (A) Hypomagnesemia; (B) hypokalemia; (C) hypocalcemia; (D) proteinuria; and (E) dehydration.
