# Supplementary material for: Safety Assessment on Serious Adverse Events of Targeted Therapeutic Agents Prescribed for RAS Wild-Type Metastatic Colorectal Cancer: Systematic Review and Network Meta-Analysis
Source: Int J Environ Res Public Health. 2022 Jul 27;19(15):9196. doi: 10.3390/ijerph19159196 (PMC9368240; doi:10.3390/ijerph19159196)
Supplement: Supplementary file 1 [file ijerph-19-09196-s001.zip › ijerph-1828077-supplementary.pdf]

Table S1. Safety ranking of biological targeting therapeutic agents

Hematological SAE

Anemia		*Febrile neutropenia*		*Neutropenia*		*Infection*		*Thrombocytopenia*	
agent	p-value	agent	p-value	agent	p-value	agent	p-value	agent	p-value
panitumumab	0.7542	cetuximab	0.8101	panitumumab	0.9055	bevacizumab	0.6783	bevacizumab	0.8444
bevacizumab	0.5559	panitumumab	0.5422	bevacizumab	0.5544	panitumumab	0.4334	cetuximab	0.5156
cetuximab	0.1899	bevacizumab	0.1477	cetuximab	0.0402	cetuximab	0.3884	panitumumab	0.14

GI SAE

Anorexia		*Nausea*		*Vomiting*		*Diarrhea*	
agent	p-value	agent	p-value	agent	p-value	agent	p-value
bevacizumab	0.6341	cetuximab	0.6463	cetuximab	0.5595	bevacizumab	0.6922
panitumumab	0.4979	panitumumab	0.6192	panitumumab	0.547	cetuximab	0.5642
cetuximab	0.3681	bevacizumab	0.2345	bevacizumab	0.3935	panitumumab	0.2436

**Neurological SAE**				**CV SAE**			
Fatigue		*Peripheral Neuropathy*		*HTN*		*Thromboembolism*	
agent	p-value	agent	p-value	agent	p-value	agent	p-value
bevacizumab	0.6418	panitumumab	0.8699	cetuximab	0.7791	cetuximab	0.7767
panitumumab	0.5926	bevacizumab	0.458	panitumumab	0.7086	bevacizumab	0.7146
cetuximab	0.2657	cetuximab	0.1718	bevacizumab	0.0123	panitumumab	0.0087

Dermatological SAE

Rash (Skin Toxicity)		*Paronychia*		*Mucositis*	
agent	p-value	agent	p-value	agent	p-value
bevacizumab	1	bevacizumab	0.9985	bevacizumab	0.9417
cetuximab	0.468	panitumumab	0.4619	cetuximab	0.5308
panitumumab	0.032	cetuximab	0.0395	panitumumab	0.0275

Renal SAE							
Hypomagnesemia		*Hypokalemia*		*Hypocalcemia*		*Overall Electrolyte abnormalities*	
agent	p-value	agent	p-value	agent	p-value	agent	p-value
bevacizumab	0.9998	bevacizumab	0.9918	cetuximab	0.8276	bevacizumab	0.9469
cetuximab	0.4886	panitumumab	0.2688	bevacizumab	0.5917	cetuximab	0.5135
panitumumab	0.0117	cetuximab	0.2394	panitumumab	0.0807	panitumumab	0.0397
Proteinuria		*Dehydration*					
agent	p-value	agent	p-value				
cetuximab	0.7961	cetuximab	0.8748				
bevacizumab	0.5381	bevacizumab	0.5934				
panitumumab	0.1658	panitumumab	0.0318				

Figure S1. Network plot of direct, indirect, network estimates of hematological SAE. A) anemia; B) febrile neutropenia; C) neutropenia; D) infection; and E) thrombocytopenia

A)

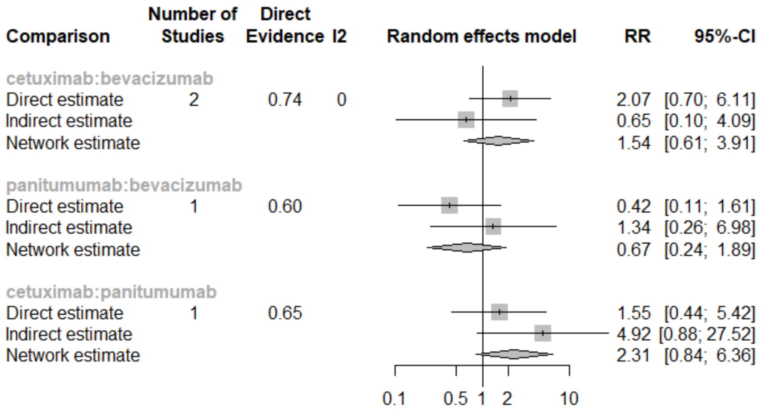

B)

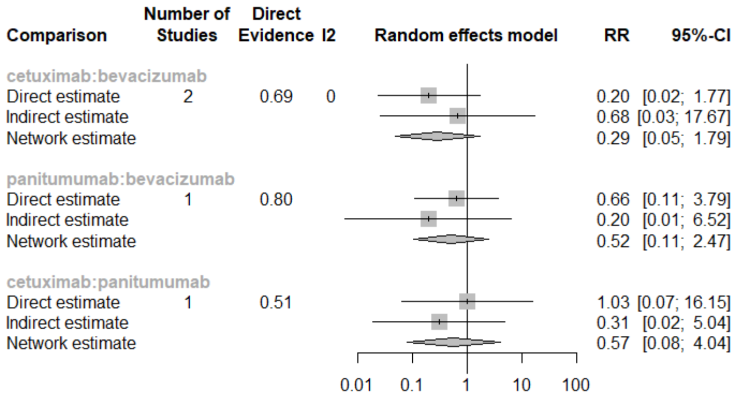

C)

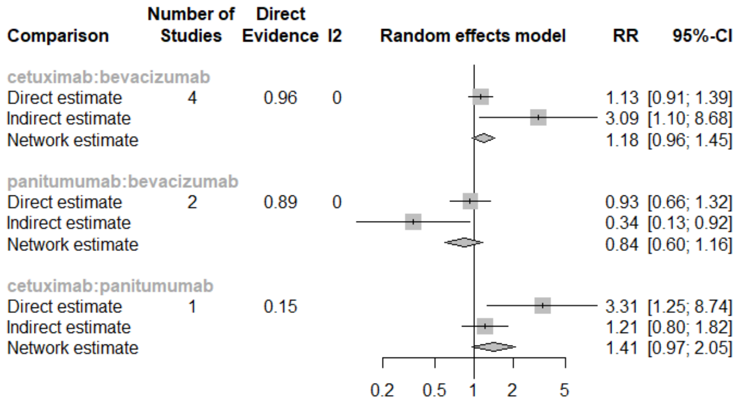

D)

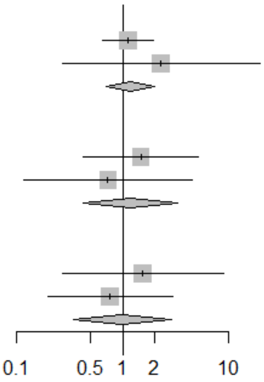

Comparison	Number of Studies	Direct Evidence	I2	RR	95%-CI
**cetuximab:bevacizumab**					
Direct estimate	1	0.94		1.13	[0.65; 1.96]
Indirect estimate				2.30	[0.27; 19.67]
Network estimate				1.18	[0.69; 2.01]
**panitumumab:bevacizumab**					
Direct estimate	2	0.69	0	1.48	[0.43; 5.11]
Indirect estimate				0.73	[0.12; 4.56]
Network estimate				1.19	[0.43; 3.31]
**cetuximab:panitumumab**					
Direct estimate	1	0.37		1.55	[0.27; 8.95]
Indirect estimate				0.76	[0.20; 2.95]
Network estimate				0.99	[0.34; 2.90]

E)

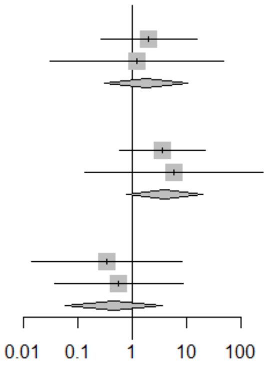

Comparison	Number of Studies	Direct Evidence	I2	RR	95%-CI
**cetuximab:bevacizumab**					
Direct estimate	2	0.76	0	2.01	[0.26; 15.31]
Indirect estimate				1.23	[0.03; 47.30]
Network estimate				1.79	[0.30; 10.55]
**panitumumab:bevacizumab**					
Direct estimate	2	0.81	0	3.55	[0.59; 21.46]
Indirect estimate				5.83	[0.13; 253.67]
Network estimate				3.89	[0.77; 19.74]
**cetuximab:panitumumab**					
Direct estimate	1	0.42		0.34	[0.01; 8.29]
Indirect estimate				0.56	[0.04; 8.52]
Network estimate				0.46	[0.06; 3.61]

Figure S2. Network plot of direct, indirect, network estimates of GI SAE. A) anorexia; B) nausea; C) vomiting; and D) diarrhea

A)

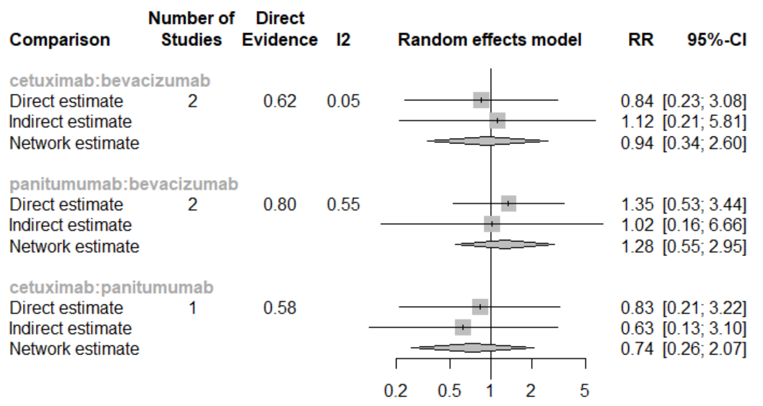

B)

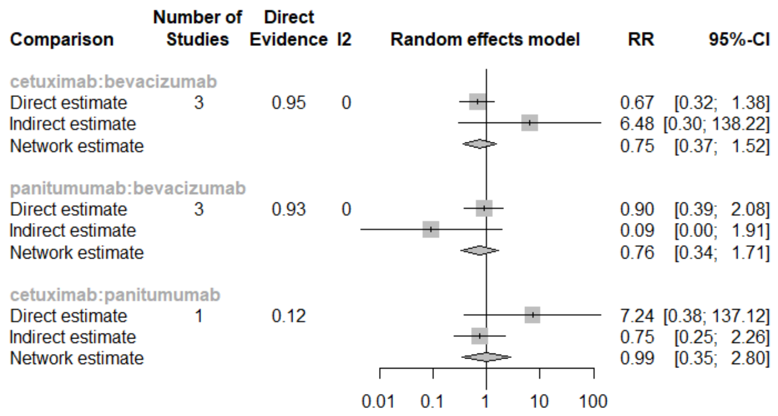

C)

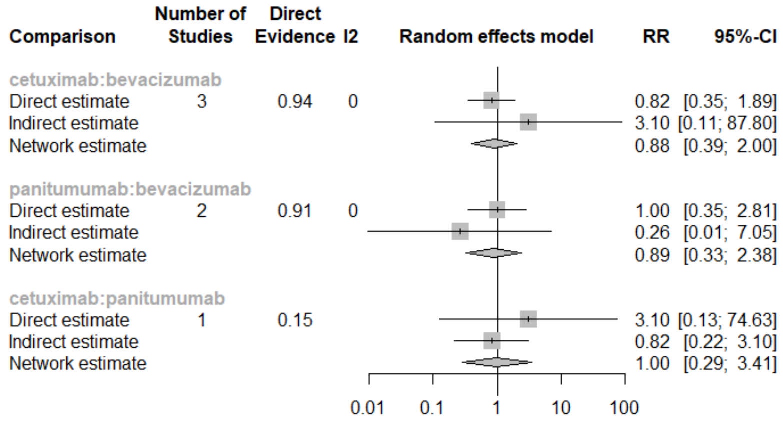

D)

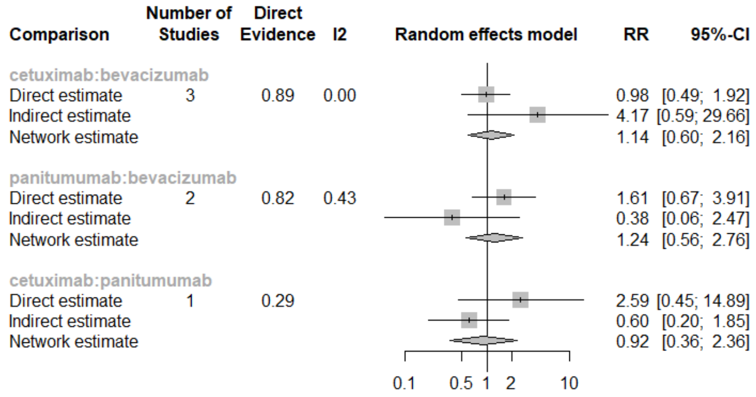
Comparison
Number of Studies
Direct Evidence
I2
Random effects model
RR
95%-CI
cetuximab:bevacizumab
Direct estimate 3 0.89 0.00 0.98 [0.49; 1.92]
Indirect estimate 4.17 [0.59; 29.66]
Network estimate 1.14 [0.60; 2.16]
panitumumab:bevacizumab
Direct estimate 2 0.82 0.43 1.61 [0.67; 3.91]
Indirect estimate 0.38 [0.06; 2.47]
Network estimate 1.24 [0.56; 2.76]
cetuximab:panitumumab
Direct estimate 1 0.29 2.59 [0.45; 14.89]
Indirect estimate 0.60 [0.20; 1.85]
Network estimate 0.92 [0.36; 2.36]
0.1
0.5
1
2
10

Figure S3. Network plot of direct, indirect, network estimates of neurological SAE. A) fatigue; and B) peripheral neuropathy

A)

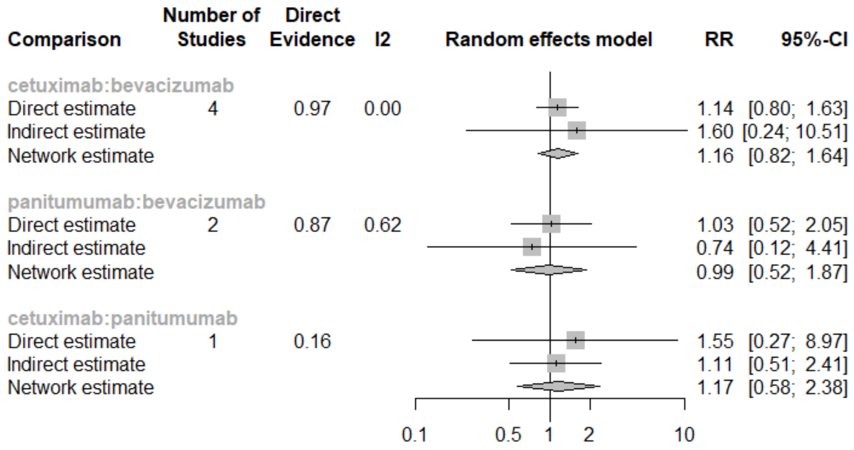

B)

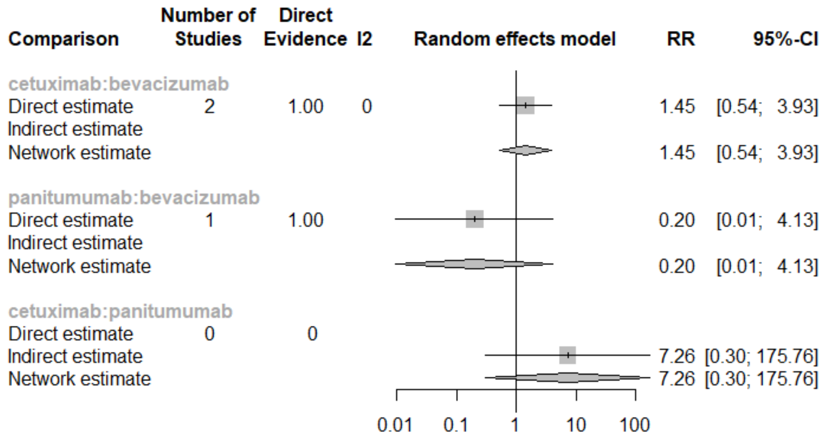

Figure S4. Network plot of direct, indirect, network estimates of CV SAE. A) HTN; and B) thromboembolism

A.

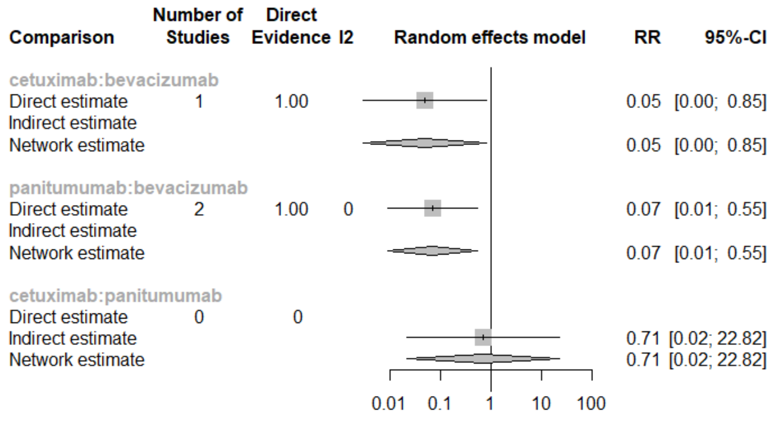

B.

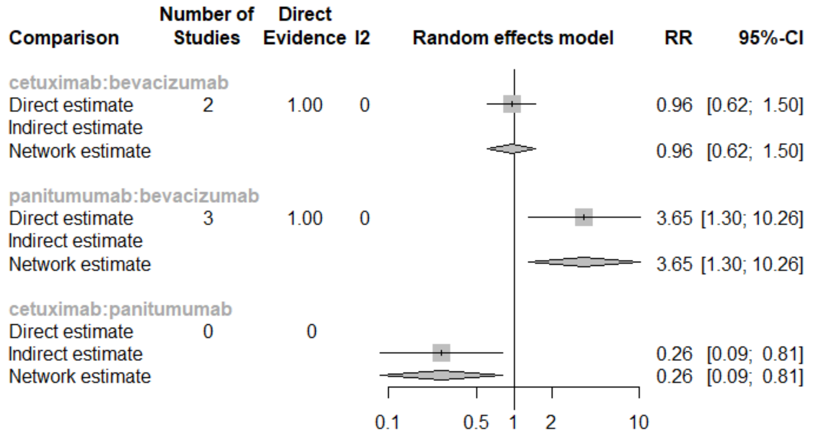

Figure S5. Network plot of direct, indirect, network estimates of dermatological SAE. A) rash (skin toxicity); B) paronychia; and C) mucositis

A.

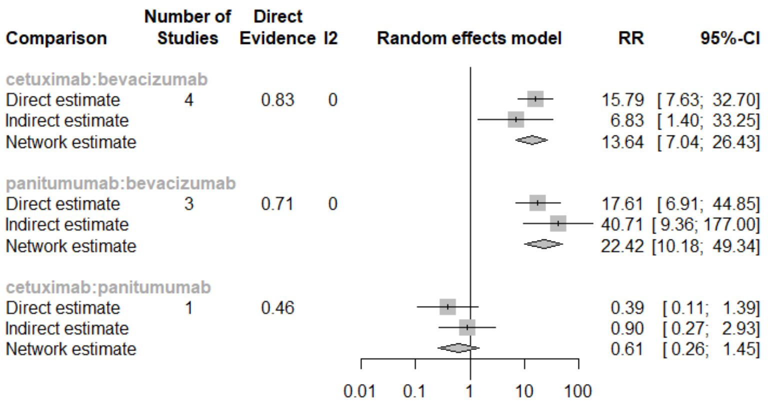

B.

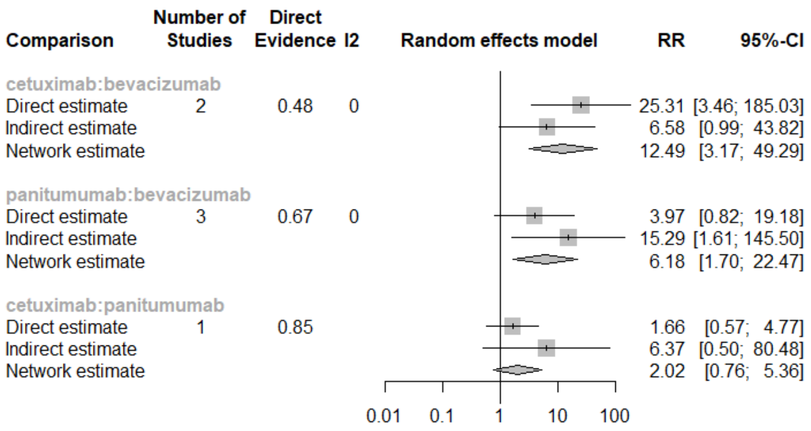

C.

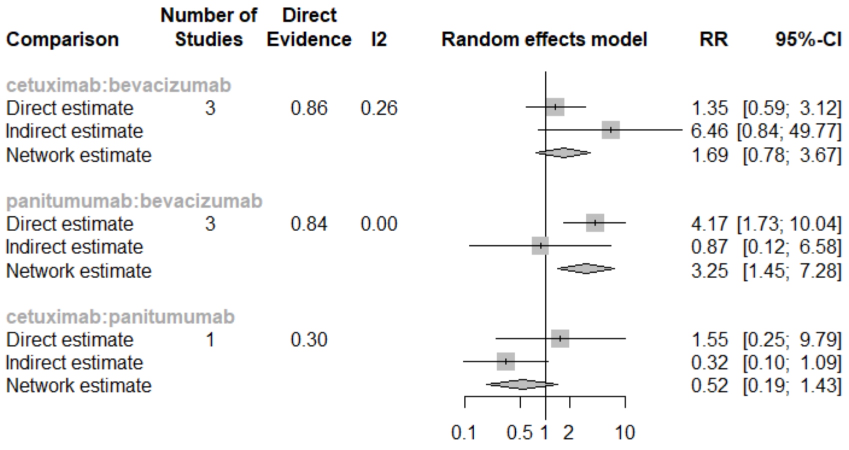

Figure S6. Network plot of direct, indirect, network estimates of renal SAE. A) hypomagnesemia; B) hypokalemia; C) hypocalcemia; D) proteinuria; and E) dehydration

A)

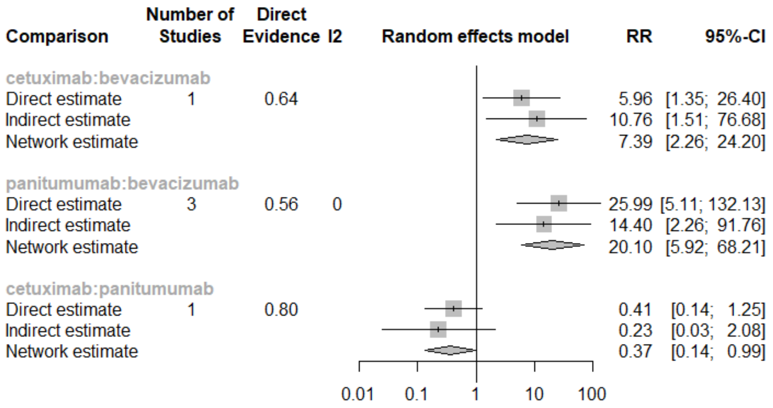

B)

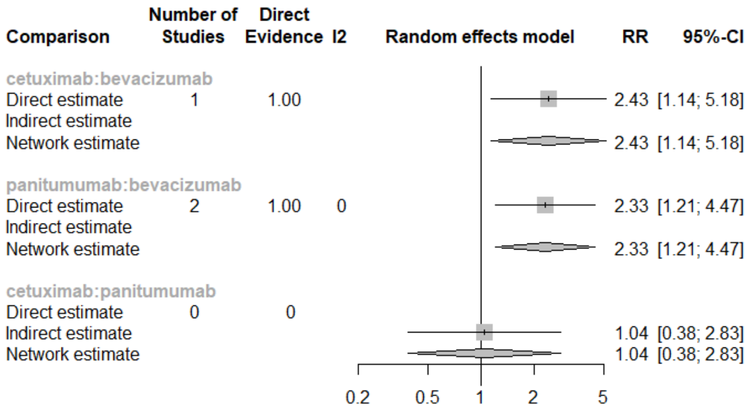

C)

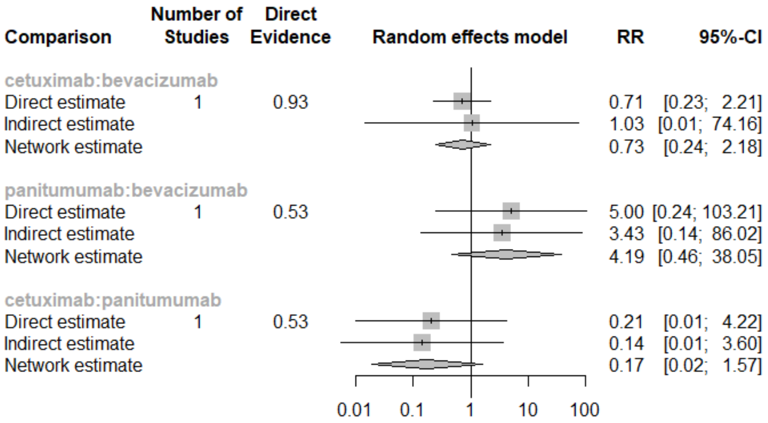

D)

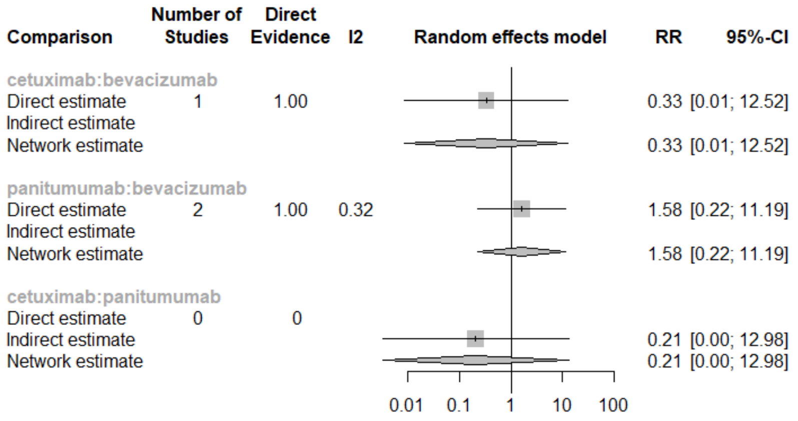
Comparison
Number of Studies
Direct Evidence
I2
Random effects model
RR
95%-CI
cetuximab:bevacizumab
Direct estimate 1 1.00 0.33 [0.01; 12.52]
Indirect estimate
Network estimate 0.33 [0.01; 12.52]
panitumumab:bevacizumab
Direct estimate 2 1.00 0.32 1.58 [0.22; 11.19]
Indirect estimate
Network estimate 1.58 [0.22; 11.19]
cetuximab:panitumumab
Direct estimate 0 0
Indirect estimate 0.21 [0.00; 12.98]
Network estimate 0.21 [0.00; 12.98]
0.01 0.1 1 10 100

E)

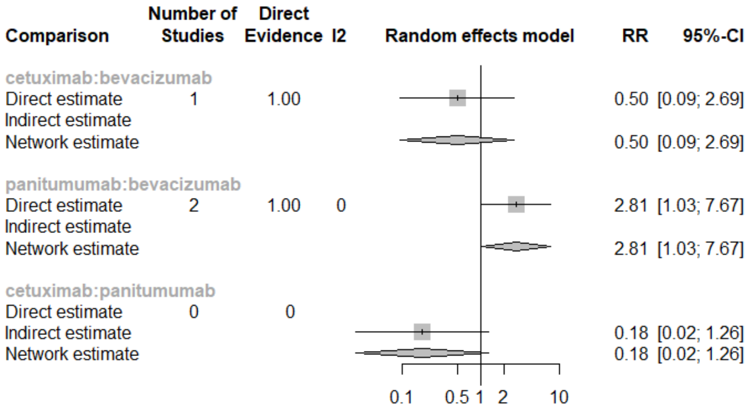
Comparison
Number of Studies
Direct Evidence
I2
Random effects model
RR
95%-CI
cetuximab:bevacizumab
Direct estimate 1 1.00 0.50 [0.09; 2.69]
Indirect estimate
Network estimate 0.50 [0.09; 2.69]
panitumumab:bevacizumab
Direct estimate 2 1.00 0 2.81 [1.03; 7.67]
Indirect estimate
Network estimate 2.81 [1.03; 7.67]
cetuximab:panitumumab
Direct estimate 0 0
Indirect estimate 0.18 [0.02; 1.26]
Network estimate 0.18 [0.02; 1.26]
0.1 0.5 1 2 10

F)

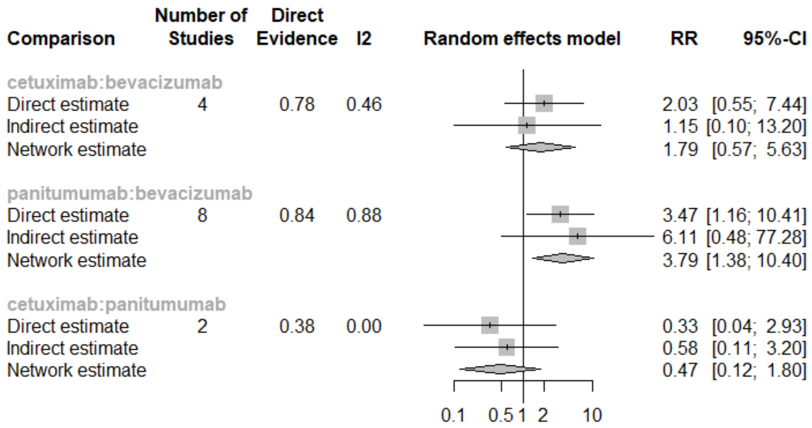
Comparison
Number of Studies
Direct Evidence
I2
Random effects model
RR
95%-CI
cetuximab:bevacizumab
Direct estimate 4 0.78 0.46 2.03 [0.55; 7.44]
Indirect estimate 1.15 [0.10; 13.20]
Network estimate 1.79 [0.57; 5.63]
panitumumab:bevacizumab
Direct estimate 8 0.84 0.88 3.47 [1.16; 10.41]
Indirect estimate 6.11 [0.48; 77.28]
Network estimate 3.79 [1.38; 10.40]
cetuximab:panitumumab
Direct estimate 2 0.38 0.00 0.33 [0.04; 2.93]
Indirect estimate 0.58 [0.11; 3.20]
Network estimate 0.47 [0.12; 1.80]
0.1 0.5 1 2 10
